# Predictive value of the Oxford Acute Severity of Illness Score in acute stroke patients with stroke-associated pneumonia

**DOI:** 10.3389/fneur.2023.1251944

**Published:** 2023-09-04

**Authors:** Ximei Wang, Jianhua Xia, Yanhua Shan, Yang Yang, Yun Li, Haiyan Sun

**Affiliations:** ^1^Department of General Critical Care Medicine, Zhumadian Central Hospital, Zhumadian, China; ^2^Department of Neurology, Zhumadian Central Hospital, Zhumadian, China; ^3^Department of Scientific Research Management, Zhumadian Central Hospital, Zhumadian, China; ^4^Department of Neurology, Jilin Province First Auto Work General Hospital, Jilin, China

**Keywords:** stroke-associated pneumonia, Oxford Acute Severity of Illness Score, stroke, diagnosis, prognosis

## Abstract

**Background:**

Stroke-associated pneumonia (SAP) is associated with a poor prognosis and a high mortality rate in stroke patients. However, the accuracy of early prediction of SAP is insufficient, and there is a lack of effective prognostic evaluation methods. Therefore, in this study, we investigated the predictive value of the Oxford Acute Severity of Illness Score (OASIS) in SAP to provide a potential reference index for the incidence and prognosis of SAP.

**Methods:**

We recruited a total of 280 patients with acute ischemic stroke who had been diagnosed and treated in the Zhumadian Central Hospital between January 2021 and January 2023. These patients were divided into an SAP group (86 cases) and a non-SAP group (194 cases) according to SAP diagnostic criteria by expert consensus on the diagnosis and treatment of SAP. We collated general and clinical data from all patients, including the survival of SAP patients during the follow-up period. Multivariate logistic regression was used to analyze the risk factors for SAP. Kaplan–Meier and multivariate COX regression analyses were used to investigate the relationship between OASIS and the prognosis of SAP, and a receiver operating characteristic (ROC) curve was drawn to analyze the predictive value of OASIS for SAP.

**Results:**

Our analyses identified body temperature, C-reactive protein, procalcitonin, OASIS, and a prolonged length of intensive care unit (ICU) stay as the main risk factors for SAP (all *P*s < 0.05). Advanced age and an elevated OASIS were identified as the main risk factors for death in SAP patients (all *P*s < 0.05). The risk of death in patients with OASIS of 31–42 points was significantly higher than that in patients with OASIS of 12–20 points (HR = 5.588, 95% CI = 1.531–20.401, *P* = 0.009). ROC curve analysis further showed that OASIS had a high predictive value for morbidity and the incidence of death in SAP patients.

**Conclusion:**

OASIS can effectively predict the onset and death of SAP patients and provides a potential reference index for early diagnosis and the prediction of prognosis in patients with SAP. Our findings should be considered in clinical practice.

## Introduction

Stroke is a common clinical disease. The results of a survey conducted in 31 provinces in China in 2020 showed the estimated prevalence (2.6%), incidence (505.2 per 100,000), and mortality (343.4 per 100,000) of stroke in people over 40 years of age in 2020, with ischemic stroke accounting for 86.8% of the total incidence (1). Stroke patients not only have to face high medical costs but also poor prognosis. According to statistics, the hospitalization cost of stroke in 2020 was as high as 58 billion yuan, of which patients paid ~19.8 billion yuan. The rate of death/discharge in hospital against medical advice was 9.2%, while ~12.5% (2.2 million people) of stroke survivors had stroke-related disability, and the disability rate at 3 and 12 months was 14.8 and 14.0%, respectively. The mortality rate of stroke at 3 and 12 months was 4.2 and 8.5%, respectively, and the recurrence rate of stroke at 3 and 12 months was 3.6 and 5.6%, respectively (2), indicating that it is urgent to improve stroke prevention and treatment strategies and strengthen assessment and early intervention in stroke prognosis in China, to improve stroke status. Stroke-associated pneumonia (SAP) is a common complication that often occurs within the 1st week after the onset of acute stroke. This condition predominantly refers to the symptoms of lung infection caused by nervous system damage and the reduced immune status that follows the onset of stroke. Previous epidemiological surveys have reported the incidence of SAP as 7–38% (3, 4). Furthermore, the risk of death within 30 days of stroke with SAP was 3-fold higher than in patients without SAP. This fact not only increases the difficulty of clinical treatment and prolongs the treatment period but also increases the incidence of severe disability (5). Over recent years, many researchers have designed SAP prediction models for different stroke patients using multivariate regression models, such as the acute ischemic stroke-associated pneumonia score (AIS-APS) and the intra cerebral hemorrhage-associated pneumonia score (ICH-APS) models, which are recommended by the Chinese Expert Consensus on the Diagnosis and Treatment of Stroke-related Pneumonia in 2019. However, the predictive accuracy of these models is not ideal, and there is a lack of predictive models to evaluate the prognosis of stroke when complicated by SAP (6, 7). Therefore, it is of great significance to continue developing early identification and accurate prognostic risk assessment methods for high-risk SAP patients to strengthen the individualized monitoring of stroke patients and implement targeted interventions to improve survival rates. Many severity scores have been used to evaluate the prognosis of critically ill patients. Of these, the acute physiology and chronic health evaluation II (APACHE II) model is the most common disease severity scoring system used in the ICU; this has had a significant effect on the prognostic evaluation of ICU patients (including those with acute stroke). However, the APACHE II system involves many parameters; most of these parameters involve laboratory tests. Furthermore, data collection can be laborious; this is not conducive to early and rapid diagnosis (8, 9). The Oxford Acute Severity of Illness Score (OASIS) is a scoring system that does not involve laboratory tests or imaging examinations. OASIS is widely used for the differential diagnosis of acute disease severity and has also been confirmed to have high identification and calibration efficiency for the prognosis of ICU patients. Consequently, OASIS can replace the more complex existing prediction systems (10, 11). However, few studies have investigated the predictive value of OASISs in the prognosis of patients with acute stroke. Moreover, nothing is known about the efficacy of OASISs when evaluating the incidence and prognosis of SAP. Therefore, in this study, we collected relevant data from patients with acute ischemic stroke and analyzed the effect of OASIS on the risk and prognosis of SAP. In addition, we investigated the predictive value of OASISs to provide reference guidelines for the prevention, treatment, and improved prognostic evaluation of patients with acute stroke complicated with SAP.

## Materials and methods

### Data sources and study population

**Figure 1:**
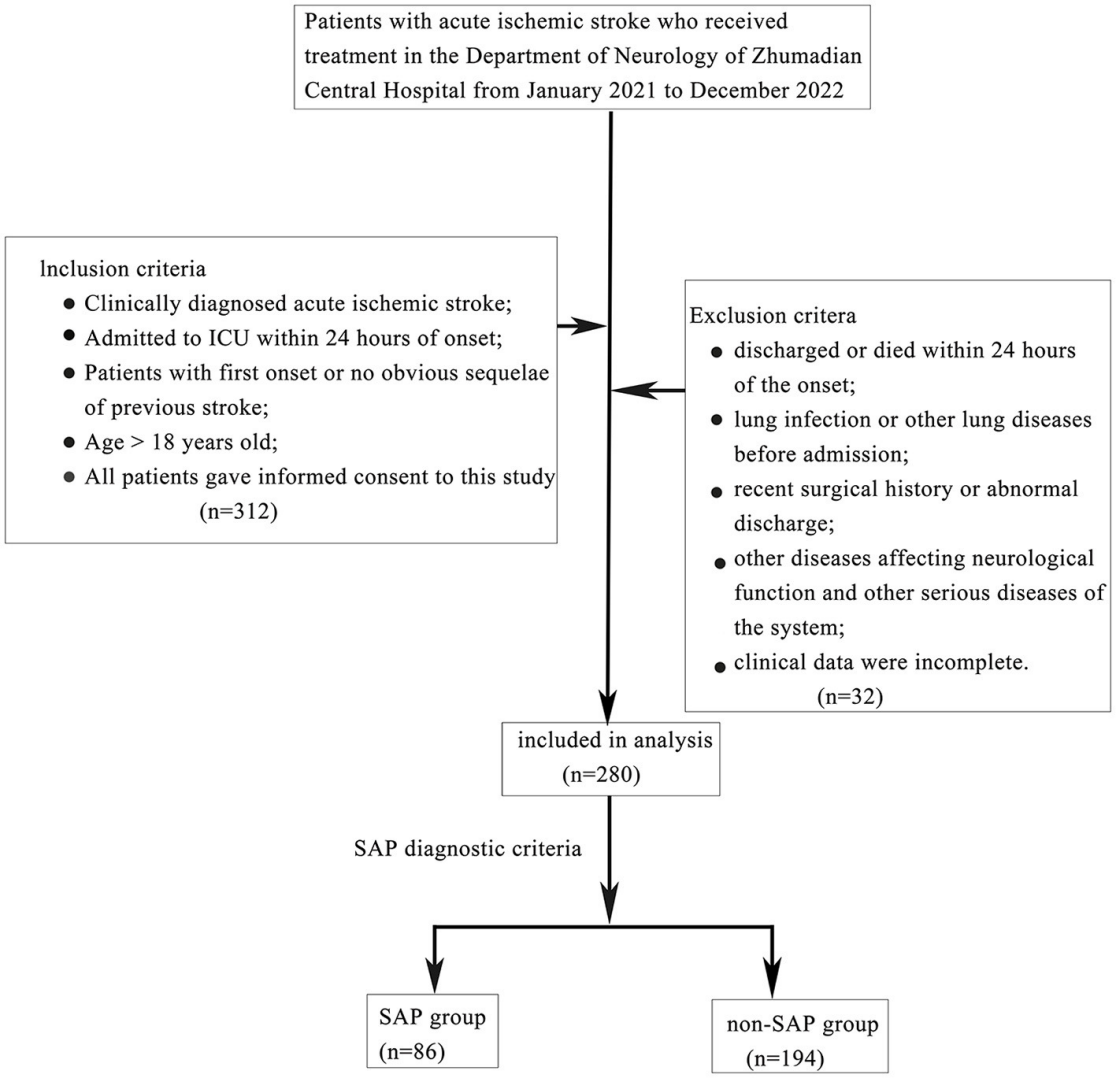
Flow chart of case collection.

We recruited patients with acute ischemic stroke who had been hospitalized in the Department of Critical Care Medicine of Zhumadian Central Hospital between January 2021 and January 2023. Patients who met the following conditions were enrolled: (1) met the diagnostic criteria for acute ischemic stroke in the 2018 Chinese Guidelines for the Diagnosis and Treatment of Acute Ischemic Stroke (12) and had been diagnosed by head computed tomography (CT) or magnetic resonance imaging (MRI); (2) admission to ICU within 24 h of onset; (3) first stroke or previous stroke without obvious sequelae; (4) aged > 18 years; and (5) provided informed consent for this study. Patients were excluded if they had been discharged within 24 h after onset or had died, had pulmonary infection or other pulmonary diseases before admission, had recent surgical history or trauma, had other diseases affecting neurological function or other serious systemic diseases, had an incomplete clinical dataset, or had been discharged abnormally. A total of 280 patients were finally included as research objects; of these, 86 SAP patients who met the SAP diagnostic criteria by the expert consensus on the diagnosis and treatment of stroke-associated pneumonia (13) were included in the SAP group; the remaining 194 patients were included in the non-SAP group (Figure 1).

We collated a range of relevant data from each patient, including demographics (age, sex, BMI, and history of past illness), vital signs on admission (heart rate, respiratory rate, mean arterial pressure, and body temperature), Glasgow coma scale (GCS) and National Institutes of Health Stroke Scale (NIHSS) scores, length of stay before ICU admission, emergency surgery before admission to the ICU, mechanical ventilation and daily urine volume on the 1st day of ICU, the length of ICU stay, complications (SAP, encephaledema, and epilepsy) and the 28-day survival of SAP patients. Laboratory tests included white blood cell count (WBC), C-reactive protein (CRP), and procalcitonin (PCT).

All patients signed informed consent forms, and this study was approved by the Medical Ethics Committee of Zhumadian Center Hospital (Approval number: 2021 - 05 - KY 001).

### Evaluations and calculations

The GCS was used to evaluate the level of consciousness of patients on the 1st day of ICU admission. The lower the score, the more serious the disturbance of consciousness. A score of 15 points indicated conscious consciousness, 13–14 points indicated a mild disturbance of consciousness, 9–12 points indicated a moderate disturbance of consciousness, 3–8 points indicated coma, and < 3 points indicated deep coma or brain death (14). The NIHSS score was used to evaluate the degree of neurological deficit in stroke patients; the higher the score, the more serious the neurological deficit; 0–1 was normal or nearly normal, 1–4 referred to mild stroke/minor stroke, 5–15 referred to moderate stroke, and 15–20 referred to moderate or severe stroke. A score of 21–42 was defined as a severe stroke (15).

The OASIS scoring system was first developed by Johnson et al. in 2013 to assess the severity of acute illness in patients without the need for laboratory indicators. Over recent years, the OASIS scoring system has been applied to the diagnosis and prognosis evaluation of acute diseases. The OASIS scoring system features 10 parameters: age, heart rate, respiratory rate, mean arterial pressure (MAP), body temperature, GCS, length of stay before ICU admission, emergency surgery prior to ICU admission, urine volume, and mechanical ventilation on the 1st day of ICU, and its scoring criteria are shown in Table 1. The total score represents the summation of each score; the higher the total score, the more severe the disease. If there were multiple records of the same item on the 1st day of ICU evaluation, the most serious record prevailed (11).

**Table 1:** Oxford Acute Severity of Illness Score.

**Item**	**Assign score**	**Item**	**Assign score**
Age (years)		Heart rate (/min)	
< 24	0	< 33	4
24–53	3	33–88	0
54–77	6	89–106	1
78–89	9	107–125	3
≥ 90	7	>125	6
Body temperature (°C)		Respiratory rate (/min)	
< 33.22	3	< 6	10
33.22–35.93	4	6–12	1
35.94–36.39	2	13–22	0
6.40–36.88	0	23–30	1
36.89–39.88	2	31–44	6
>39.88	6	>44	9
Mean arterial pressure (mmHg)		Length of stay before ICU admission (h)	
< 20.65	4	< 0.17	5
20.65–50.99	3	0.17–4.94	3
51.00–61.32	2	4.95–24.00	0
61.33–143.44	0	24.01–311.80	2
>143.44	3	>311.80	1
Emergency surgery prior to ICU admission		Mechanical ventilation on the first day of ICU	
No	0	No	0
Yes	6	Yes	9
Urine volume on the first day of ICU (ml)		GCS	
< 671		3–7	10
671–1,426		8–13	4
1,427–2,543		14	3
2,544–6,896		15	0
>6,896			

### Follow-up and endpoints

Patients with SAP were followed-up for 28 days (the 1st day of follow-up was the day of SAP diagnosis daily). The endpoints for SAP patients included progression-free survival (PFS) and overall survival (OS). PFS was defined as the time from the beginning of SAP intervention to the observation of disease progression or death from any cause. OS was defined as the time from SAP diagnosis to the occurrence of death from any cause.

### Statistical analysis

All data were analyzed using SPSS (version 26.0) statistical software. The Shapiro–Wilk test was used to evaluate the normality of measurement data; if the data conformed to the normal distribution, then the mean ± standard deviation (SD) are given, and the independent sample *t*-test was used to compare the two groups. Measurement data that were not normally distributed are expressed as medians and inter-quartile ranges (IQR), and the Mann–Whitney test was used to compare the two groups. Count data are expressed as a frequency and percentage, and the chi-square (χ^2^) test was used to compare the two groups. Multiple logistic regression was used to analyze the influencing factors of patients with acute stroke complicated with SAP. A Kaplan–Meier survival curve was used to analyze the correlation between OASISs, PFS, and OS in SAP patients, and multiple COX regression was used to analyze the factors that influence prognosis and survival in SAP patients. Receiver operating characteristic (ROC) curve analysis was used to analyze the value of OASISs to predict the incidence and prognosis of SAP. A *P*-value of < 0.05 was considered to indicate statistical significance.

## Results

### Comparison of general clinical data between the non-SAP and SAP groups

Table 2 depicts the analysis of general and clinical data from patients in the SAP and non-SAP groups. There were no significant differences between the two groups with regard to gender, age, BMI, past medical history, heart rate, respiratory rate, mean arterial pressure, GCS score, NIHSS score, emergency operation before ICU admission, urine volume on the 1st day of ICU admission, length of stay before ICU admission, proportion of encephaledema and epilepsy, and WBC upon admission (all *P* > 0.05). However, age, body temperature, the levels of CRP and PCT, and OASIS at admission were significantly higher in the SAP group when compared to the non-SAP group (*Z* = 2.158, *P* = 0.031; *Z* = 4.410; *Z* = 4.410, *P* = 0.000; *t* = 3.350, *P* = 0.001; *t* = 2.973, *P* = 0.003; *t* = 7.017, *P* = 0.000). Furthermore, the length of ICU stay was significantly longer in the SAP group (*Z* = 2.338, *P* = 0.000).

**Table 2:** Comparison of general and clinical data between non-SAP group and SAP group.

**Variables**	**Non-SAP (*n* = 194)**	**SAP (*n* = 86)**	**Test value**	***P*-value**
**Sex**, ***n*** **(%)**				
Male	77 (39.69)	36 (41.86)	*χ^2^*= 0.117	0.733
Female	117 (60.31)	50 (58.14)		
Age (years), median (IQR)	52 (43.75, 66.00)	58.5 (28.50, 66.25)	*Z* = 2.158	0.031
BMI (kg/m^2^), mean (±SD)	23.82 (±2.03)	24.31 (±2.46)	*t* = 1.734	0.084
**History of past illness**, ***n*** **(%)**				
Yes	94 (48.45)	36 (41.86)	*χ^2^*= 1.041	0.308
No	100 (51.55)	50 (58.14)		
Heart rate (/min), median (IQR)	81.00 (55.00, 112.25)	91.50 (55.00, 122.00)	*Z* = 0.658	0.510
Respiratory rate (/min), median (IQR)	24.00 (15.00, 35.25)	22.00 (13.75, 31.50)	*Z* = 1.076	0.282
MAP (mmHg), median (IQR)	81.50 (54.75, 117.00)	81.00 (47.75, 100.75)	*Z* = 0.996	0.319
Body temperature (°C), median (IQR)	37.90 (37.00, 39.30)	39.30 (38.00, 40.20)	*Z* = 4.410	0.000
GCS (score), median (IQR)	9.00 (7.00, 12.00)	7.00 (5.75, 12.00)	*Z* = 1.484	0.052
NIFSS (score), mean (±SD)	20.48 (±9.54)	21.05 (±9.31)	*t* = 0.462	0.644
**Emergency surgery prior to ICU admission**, ***n*** **(%)**				
Yes	90 (46.39)	49 (56.98)	*χ^2^* = 2.671	0.102
No	104 (53.61)	37 (43.02)		
**Mechanical ventilation on the first day of ICU**, ***n*** **(%)**				
Yes	44 (22.68)	0 (0.00)	–	–
No	150 (77.32)	86 (100.00)		
Urine volume on the first day of ICU (ml), mean (±SD)	3,918.35 (±1,563.13)	3,652.81 (±1,902.52)	*t* = 1.224	0.222
Length of stay before ICU admission (h), mean (±SD)	11.71 (±7.05)	10.89 (±6.99)	*t* = 0.897	0.370
length of ICU stay (day), median (IQR)	13.00 (10.00, 18.00)	19.00 (13.00, 28.00)	*Z* = 2.338	0.000
**Complication**, ***n*** **(%)**				
Encephaledema	19 (9.79)	8 (9.30)	*χ^2^* = 0.017	0.898
Epilepsy	4 (2.06)	0 (0.00)	*χ^2^* = 1.799	0.180
WBC (× 10^9^/L), median (IQR)	16.90 (15.22, 21.82)	17.14 (13.82, 20.87)	*Z* = 0.840	0.480
CRP (mg/L), mean (±SD)	10.77 (±4.68)	12.83 (±4.87)	*t* = 3.350	0.001
PCT (ng/mlL), mean (±SD)	1.89 (±0.63)	2.18 (±0.99)	*t* = 2.973	0.003
OASIS (score), mean (±SD)	19.81 (±6.33)	25.69 (±6.74)	*t* = 7.017	0.000

History of past illness including hypertension, hyperlipidemia, diabetes, stroke, atrial fibrillation, etc.; mechanical ventilation on the first day of ICU: only descriptive statistics, no comparison of differences between the two groups because of that SAP was defined as the new symptoms of pulmonary infection in stroke patients who were not mechanically ventilated within 7 days of onset. IQR, inter-quartile ranges; SD, standard deviation; BMI, body mass index; MAP, mean arterial pressure; GCS, Glasgow coma Scale; NIFSS, National Institute of Health stroke scale; ICU, intensive care unit; WBC, white blood cell count; CRP, C-reactive protein; PCT, procalcitonin; OASIS, Oxford Acute Severity of Illness Score.

### Analysis of risk factors for acute stroke patients complicated by SAP

SAP occurred was defined as the dependent variable (yes = 1, no = 0) and multiple logistic regression analysis was conducted using age, body temperature, CRP and PCT levels, OASIS, and length of ICU stay as independent variables. Sex, BMI, history of past illness, heart rate, respiratory rate, MAP, GCS, NIHSS score, emergency surgery before ICU admission, urine volume on the 1st day of ICU admission, length of stay before ICU admission, proportion of encephaledema and epilepsy, and WBC on admission were utilized as covariates. Analysis showed that the risk factors for acute stroke patients with SAP in acute stroke patients were body temperature, CRP and PCT levels, OASIS, and length of ICU stay at admission. The higher the levels of CRP and PCT, the higher the risk of SAP [odds ratio (OR) = 1.611, *P* < 0.001; OR = 1.081, *P* = 0.049; OR = 1.685, *P* = 0.023]; furthermore, a higher OASIS indicated a higher risk of SAP (OR = 1.136, *P* < 0.001) (Table 3).

**Table 3:** Multivariate logistic regression analysis of acute stroke patients complicated with SAP.

	**Odds ratio (OR)**	**95% CI**	***P*-value**
Age	1.013	0.975–1.040	0.689
Body temperature	1.611	1.265–2.051	< 0.001
CRP	1.081	1.000–1.168	0.049
PCT	1.685	1.073–2.645	0.023
OASIS	1.136	1.076–1.200	< 0.001
Length of ICU stay	1.128	1.077–1.182	< 0.001
Sex (male vs. female)	1.319	0.666–2.612	0.426
BMI	1.080	0.927–1.258	0.324
History of past illness (yes vs. no)	0.860	0.434–1.703	0.666
Heart rate	0.999	0.990–1.008	0.827
Respiratory rate	0.975	0.946–1.006	0.112
MAP	0.997	0.987–1.007	0.539
GCS	0.931	0.843–1.028	0.155
NIFSS	0.993	0.956–1.031	0.708
Emergency surgery prior to ICU admission	0.843	0.414–1.719	0.639
Urine volume on the first day of ICU	1.000	1.000–1.000	0.431
Length of stay before ICU admission	0.991	0.943–1.042	0.726
Encephaledema (yes vs. no)	0.487	0.155–1.533	0.219
WBC	1.041	0.968–1.120	0.282

### Predictive diagnosis of acute stroke patients with SAP

**Figure 2:**
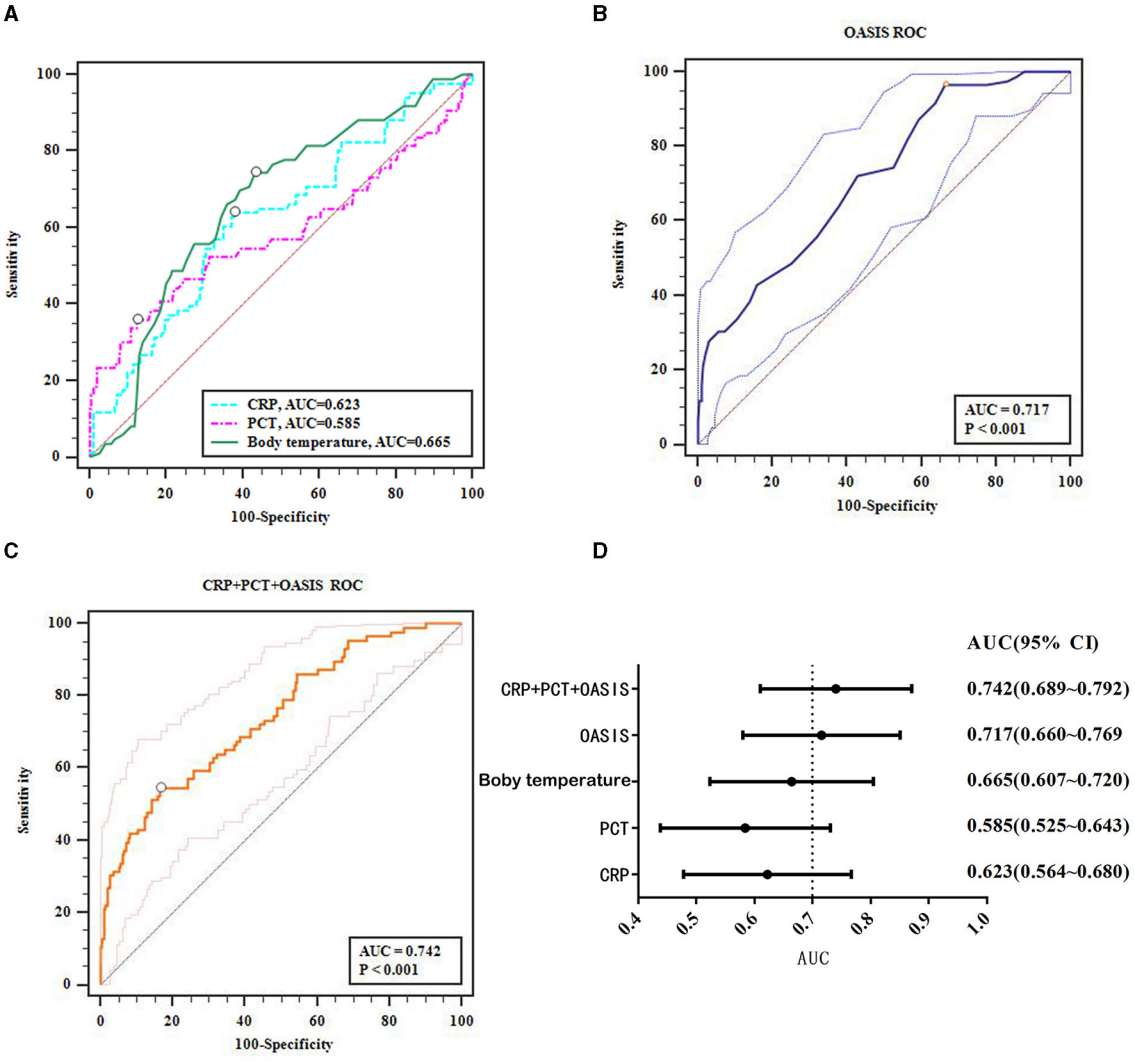
ROC curves analyze the predictive value of the major factors that affect the occurrence of SAP. **(A)** ROC results for CRP, PCT, temperature; **(B)** OASIS ROC; **(C)** CRP + PCT + OASIS ROC; **(D)** AUC of the major factors that affect the occurrence of SAP.

Multiple logistic regression analysis found that body temperature, CRP and PCT levels, and OASIS at admission were all risk factors for SAP in acute stroke patients. Next, we considered whether these factors could hold predictive value for SAP. ROC curve analysis showed that body temperature, and CRP and PCT levels had an average predictive value for SAP in acute stroke patients (AUCs of 0.665, 0.623, and 0.585, respectively; Figure 2A), while OASIS had a high predictive value (AUC: 0.717, Figure 2B). To improve the predictive value of OASIS, we next analyzed the combined predictive value of OASIS + CRP + PCT and found that the predictive value of this combination of parameters (AUC: 0.742, Figure 2C) was higher than that for OASIS; however, this was not significant (*Z* = 1.558, *P* = 0.119). The 95% confidence interval range of the AUCs for each variable is shown in Figure 2D.

### Relationship between OASIS and the prognosis of patients with acute stroke complicated with SAP

**Figure 3:**
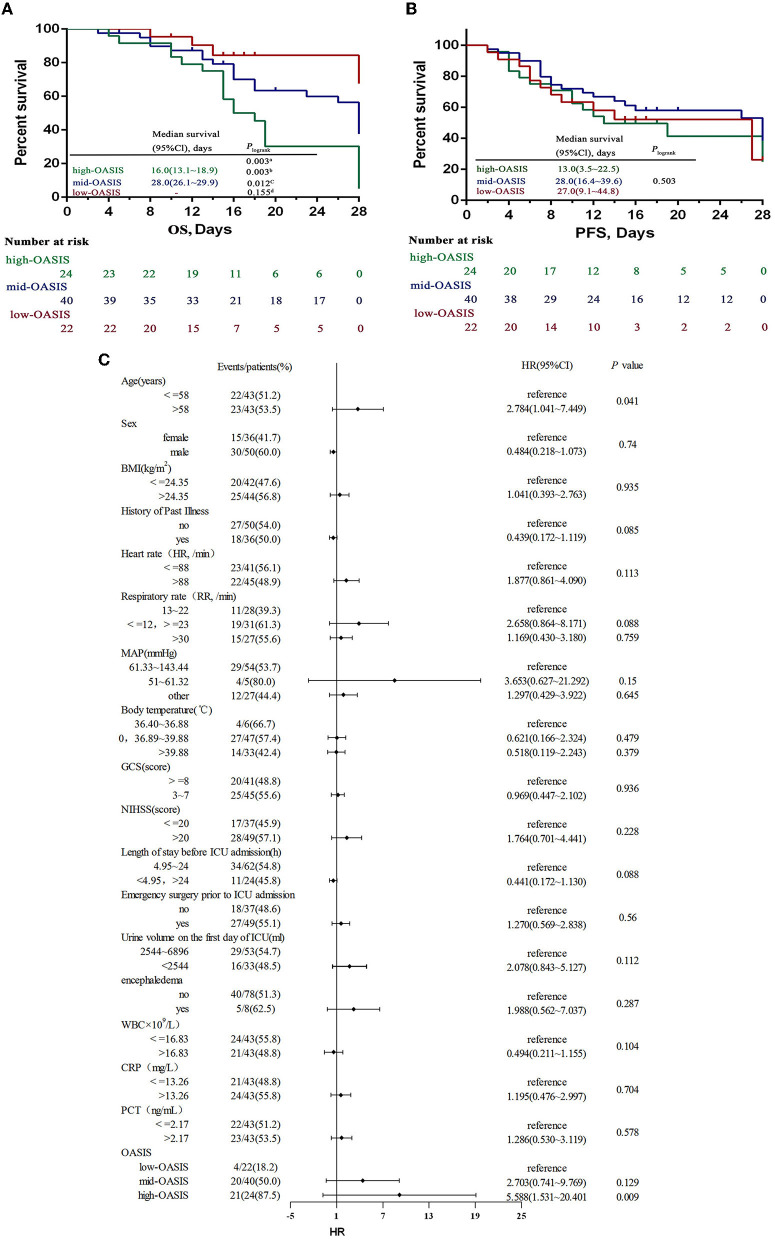
OS **(A)** and PFS **(B)** by different OASIS groups and ROC curves **(C)** for OASIS. OS overall survival, PFS progression-free survival. ^a^Three-group comparison: *P* < 0.05, ^b^Comparison between high-OASIS and low-OASIS: *P* < 0.05, ^c^Comparison between high-OASIS and med-OASIS: *P* < 0.05, ^d^Comparison between med-OASIS and low-OASIS: *P* < 0.05. -The analyses failed because of <50% died in the low-oasis group.

The highest and lowest OASISs for the 86 SAP patients were 42 points and 12 points, respectively; these scores were divided into three groups according to the score range: a high-OASIS group (31–42 points), a mid-OASIS group (21–30 points), and a low-OASIS group (12–20 points) (16). Kaplan–Meier analysis showed that the OS of the high-OASIS group was significantly shorter than that of the mid-OASIS group and the low-OASIS group (*P* = 0.012; *P* = 0.003) and that there was no significant difference between the mid-OASIS and low-OASIS groups (*P* = 0.155) (Figure 3A), thus indicating that OASIS was related to OS. However, there was no significant difference in PFS among the three groups (*P* = 0.503) (Figure 3B); thus, OASIS was not correlated with PFS.

### OASIS has an independent effect on OS in SAP patients

A COX regression model was established to analyze the risk factors for prognosis and survival in patients with acute stroke complicated by SAP. Death was used as the state variable and OS was used as the time variable; various other factors were used as covariates: age, sex, BMI, history of past illness, heart rate, respiratory rate, MAP, body temperature, GCS, NIHSS, length of stay before ICU admission, emergency surgery prior to ICU admission, urine volume on the 1st day of ICU, encephaledema, WBC, CRP and PCT levels, and OASIS. Prior to COX regression analysis, the continuity covariates were transformed into categorical variables; age, BMI, WBC, and CRP and PCT levels were demarcated by the median value. Furthermore, heart rate, respiratory rate, MAP, body temperature, GCS, length of stay before ICU admission, and urine volume on the 1st day of ICU were classified by referring to the scoring standards of various parameters in the OASIS model; NIHSS classification refers to the NIHSS grading standards. Analysis showed that age and OASIS were independent risk factors that affect the prognostic OS in SAP patients (*P* = 0.041; *P* < 0.001); the risk of death was 2.784-fold higher in patients over 58 years of age than in patients under 58 years of age (95% CI: 1.041–7.449), 5.588-fold higher in patients in the high-OASIS group than those in the low-OASIS group (95% CI: 1.531–20.401), and 2.703-fold higher in patients in the mid-OASIS group than in the low-OASIS group (95% CI: 0.741–9.769) (Figure 3C). These data proved that OASIS was associated with SAP prognosis and that an elevated OASIS was a major independent risk factor for death in patients with SAP.

### Prognostic diagnosis of OASIS in SAP patients

**Figure 4:**
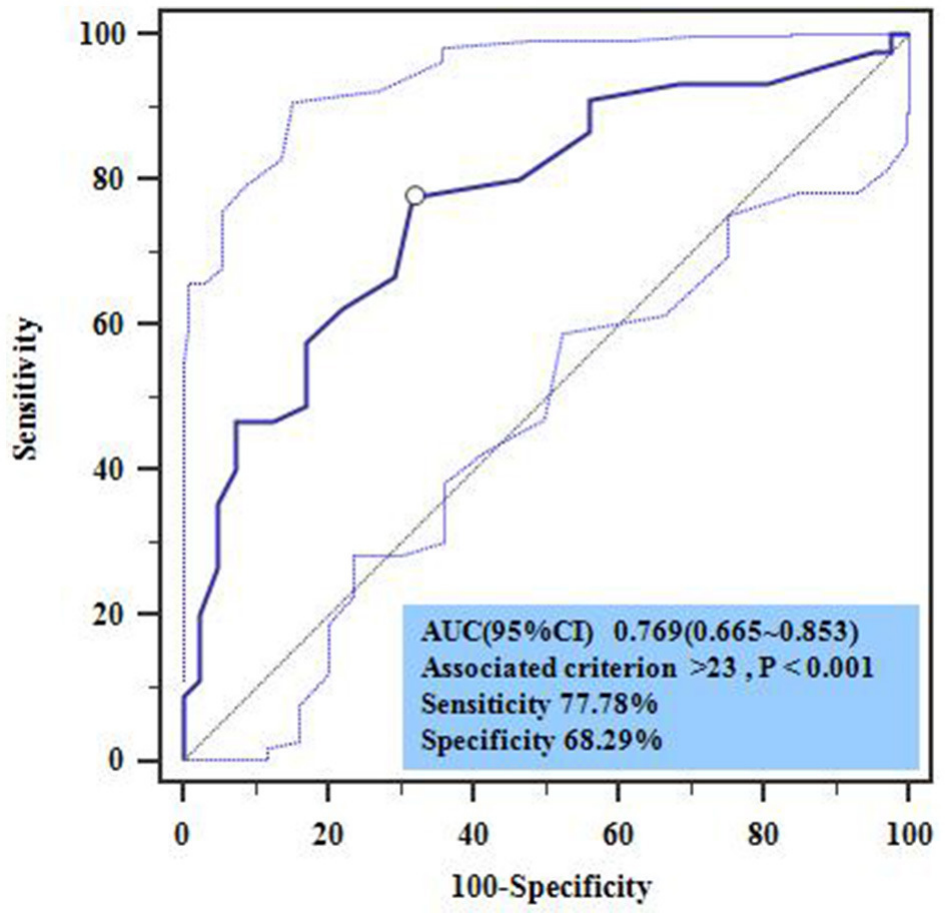
Multivariate cox regression analysis of prognostic death in SAP patients. HR, hazard ratio; high-OASIS 31–42 scores; med-OASIS 21–30 scores; low-OASIS 12–20 scores.

ROC curve analysis was used to explore the diagnostic value of OASIS in predicting the death of SAP patients. Analysis showed that OASIS had a high predictive diagnostic value (AUC = 0.769, *P* < 0.001) with a sensitivity of 77.88% and a specificity of 68.29% (Figure 4).

## Discussion

SAP is a serious complication in stroke patients, which can affect the prognosis of patients and increase the risk of disability and death, thus generating a heavy burden for both family and society. Although previous researchers have investigated the early diagnosis of SAP, there is no consensus of opinion. Furthermore, these existing prediction methods differ widely, thus reducing the accuracy of early SAP prediction and the lack of prevention or timely anti-infection treatment, thus increasing the risk of adverse events. Therefore, actively investigating early diagnosis and prognostic risk assessment methods for SAP is a key strategy to reduce the incidence of adverse prognostic events in stroke.

In the present study, we analyzed general and clinical data from patients in the SAP group and the non-SAP group. Analysis showed that age, body temperature, CRP and PCT levels, and OASISs of patients in the SAP group were significantly higher than those in the non-SAP group; furthermore, the length of ICU stay was longer in the SAP group. CRP and PCT levels, and OASIS were identified as the main risk factors for patients with ischemic stroke complicated by SAP, thus indicating that the onset of SAP is significantly affected by the patient's age, body temperature at admission, CRP and PCT levels, and OASIS. As age increases, organ function and immunity decline, thus making the body vulnerable to invasion by exogenous substances; this could increase the risk of pulmonary infection (17). However, stroke is often accompanied by an inflammatory response that could lead to an imbalance in the system that regulates body temperature; this may increase body temperature, reduce immunity, and increase the risk of pathogenic infection and invasion of the lungs, thus aggravating the inflammatory response and immune response disorders. Collectively, these events may lead to a further increase in body temperature and the levels of CRP and PCT, thus increasing the risk of pneumonia (18). OASIS is a comprehensive evaluation index based on 10 parameters, including age, body temperature, and urine volume on the 1st day of ICU. The higher the OASIS, the more severe the disease. Stroke patients with more severe diseases have relatively poor body and immune function, thus increasing the risk of pneumonia. In a previous study, Szylińska et al. showed that advanced age, elevated CRP level, and prolonged hospital stay were all associated with the onset of SAP and represented independent risk factors for SAP (19). In another study, Kuo et al. showed that age, body temperature, pulse rate, and NIHSS score on admission were the main predictors of post-stroke pneumonia (20). Other studies have also pointed out that the older the patient, the higher the incidence of SAP; preoperative neutrophil count, lymphocyte count, and the levels of PCT and CRP were all identified as risk factors for SAP, thus indicating that PCT and CRP can be used as early predictive indicators of SAP (21, 22), thus supporting our current findings. However, OASIS has rarely been included in the multivariate regression analysis of SAP in previous studies. This may be because OASIS is widely recommended to evaluate acute conditions, but less attention has been paid to its application for acute complications. However, several parameters in the OASIS model, including age, body temperature, and heart rate, have been confirmed by most researchers to be closely related to the incidence of SAP, thus indicating that OASISs may affect the incidence of SAP. Our current findings confirmed that OASISs are closely related to the risk of SAP. It is worth noting that the effect of age on the incidence of SAP in our logistic regression analysis was not obvious; it is possible that this was related to our small sample size or interactions between OASIS and age.

Based on the risk factors of SAP, many researchers have attempted to establish SAP risk models using a range of risk factors, including the A2D2 score scale, the preventive antibacterial therapy in acute ischemic stroke score scale, and the AIS-APS scale (5). However, these scoring systems have limitations that need to be considered. For example, these systems can only be applied in a limited number of scenarios. Furthermore, some scoring systems involve a number of laboratory parameters, thus creating limitations for early rapid prediction. The OASIS model is widely used in the ICU because it is applicable to all types of acute diseases and does not involve laboratory indicators; however, its diagnostic efficiency can vary. A previous study reported that the OASIS model exhibited a good discriminative value to differentiate qSOFA (quick sequential organ failure assessment)-negative patients with sepsis from those without sepsis (AUC: 0.753; 95% CI: 0.741–0.765) (23). Another study reported the general diagnostic value of the OASIS model for sepsis after cardiac surgery (AUC: 0.650; 95% CI: 0.638–0.661) (24). In this study, ROC curve analysis was used to analyze the predictive value of body temperature, PCT and CRP levels, and OASIS for SAP; analysis revealed a general predictive value for body temperature, and PCT and CRP levels (AUCs: 0.665 0.585, 0.623; 95% CIs: 0.607–0.720, 0.525–0.643, 0.564–0.680); OASIS had a higher value for predicting SAP (AUC: 0.717; 95% CI: 0.660–0.769). In addition, we also analyzed the combined predictive value of OASIS + CRP + PCT and found that the predictive value of OASIS + CRP + PCT (AUC: 0.742; 95% CI: 0.889–0.792) was higher than that of OASIS alone although this was not significant (*Z* = 1.558, *P* = 0.119). Considering the clinical practice, we believe that although the combination of OASIS + CRP + PCT improves the diagnostic value partly, it takes a long time and is not conducive to rapid diagnosis because it involves laboratory indicators and needs to collect CRP and PCT of patients. Therefore, OASIS can predict the risk of SAP and represents an objective and simple model that does not require biochemical parameters and is worthy of clinical promotion.

Other studies have shown that SAP patients often have a worse prognosis, such as prolonged hospital stay and significantly increased hospital mortality, thus exerting a significant impact on medical resources and the economy. At present, the early prediction of SAP prognosis is of great significance for formulating early intervention measures, improving prognosis, and reducing mortality (25, 26). However, the current research on the prognostic value of stroke complicated with SAP was limited to laboratory indicators (27), which brought a lot of work to the assessment and difficult to make an early assessment quickly and accurately, which may delay the intervention. Therefore, a concise, rapid, and accurate stroke prognosis assessment method is of great significance for reducing the adverse prognosis of stroke patients. As an important reference index for the differential diagnosis of acute diseases, the OASIS model not only has a high value for the differential diagnosis of patient conditions but also has a certain reference value for predicting the prognosis of acute diseases. In a previous study, Chen et al. showed that OASIS was significantly correlated with the hospital mortality of sepsis patients (including patients with stroke-related sepsis) (OR: 1.07; 95% CI: 1.06–1.08) and had a certain ability to predict death (AUC: 0.652; 95% CI: 0.636–0.668) (28). Zhu et al. showed that OASIS had a high predictive value for 28-day mortality in patients with sepsis (AUC: 0.753; 95% CI: 0.742–0.764) (29), and it was also associated with death in machine-ventilated stroke patients (*P* < 0.001) (30). Huang et al. found that OASIS was correlated with the prognosis of ICU patients with respiratory failure (including patients with stroke-related respiratory failure) (OR: 1.06; 95% CI: 1.05–1.08) and also had some predictive value for prognostic mortality (AUC: 0.664; 95% CI: 0.644–0.685) (31). However, the current studies for OASIS in stroke prognosis lack pertinency, and the predictive value for stroke with SAP prognosis has not been reported previously. In the present study, survival analysis was used to analyze the relationship between OASISs and the survival rates of SAP patients; analysis showed that OASIS was not related to PFS but was related to OS. Patients with an OASIS of 31–42 had a significantly shorter OS than those with a score of 21–30 and 12–20 (*P* < 0.05). To further verify the relationship between OASIS and OS, multiple COX regression analysis was performed by adding confounding factors, such as age and sex; results showed that age and OASIS were independent risk factors for the OS of SAP patients. The mortality risk for SAP patients older than 58 years was significantly higher than that for patients younger than 58 years (OR: 2.784; 95% CI: 1.041–7.449). The mortality risk of SAP patients with an OASIS of 31–42 was 5.588-fold higher than those with scores of 12–20 scores (95% CI: 1.531–20.401). Patients with scores of 21–30 had a 2.703-fold higher risk than those with scores of 12–20 (95% CI: 0.741–9.769). These findings suggested that OASIS is closely related to death in SAP patients and that increasing age elevates the risk of death in SAP patients. Because the OASIS system includes age parameters, this study only considered the predictive diagnostic value of OASIS in terms of the death of SAP patients. ROC curve analysis showed that OASIS had a high predictive value (AUC: 0.769; 95% CI: 0.665–0.853) with a sensitivity of 77.88% and a specificity of 68.29%; the best cutoff value of OASIS for predicting death was 23 points. Thus, we have confirmed that OASIS can predict the prognosis death of SAP patients efficiently and also suggests that clinical attention should be given to SAP patients aged >58 years; in such patients, early intervention measures should be actively taken to reduce the incidence of a poor prognosis.

However, our study has some limitations that need to be considered. First, the subjects in this study had ischemic stroke and had been treated in the General Intensive Care Department of Zhumadian Central Hospital; thus, our data are not fully representative of the general population. Second, the cases in this study were all patients with cerebral ischemic stroke patients; thus, we only considered a specific condition with limited applicability. Third, the sample size was insufficient, with only 86 SAP patients and 24 high-OASIS patients; this may be the reason for the high mortality rate of SAP. These deficiencies need to be addressed in future research.

## Conclusion

The analysis demonstrated that OASIS can effectively predict the onset and death of patients with SAP and provide a potential reference index for early diagnostic and prognostic predictions for patients with SAP that can readily be translated to the clinic. Our findings have great significance in the prevention of SAP in patients with ischemic stroke, thus increasing survival rates and improving prognosis.

## Data availability statement

The raw data supporting the conclusions of this article will be made available by the authors, without undue reservation.

## Ethics statement

The studies involving humans were approved by Medical Ethics Committee of Zhumadian Central Hospital. The studies were conducted in accordance with the local legislation and institutional requirements. The participants provided their written informed consent to participate in this study.

## Author contributions

XW contributed to the conception or design of the work, acquisition, analysis, and interpretation of data for the work. HS guided article design, drafting, the work, and revised it critically for important intellectual content. JX and YL contributed to the case collection and experiments operation. YS and YY contributed to the manuscript final revision and supervision. All authors contributed to the article and approved the submitted version.
